# Invisible harm and constrained response: a conceptual analysis of psychological domestic abuse against disabled people in China

**DOI:** 10.3389/fpsyg.2026.1928590

**Published:** 2026-08-24

**Authors:** Zehua Feng, Yilin Li, Wenzong Xia, Yu Hu

**Affiliations:** 1 Institute for National Security and Human Rights, Guangdong University of Technology, Guangzhou, China; 2 Law School, Hubei University, Wuhan, China

**Keywords:** China, coercive control, disabled people, evidence, help-seeking, psychological domestic abuse, structural alignment

## Abstract

Psychological domestic abuse against disabled people is under-recognised at the intersection of violence prevention, public mental health, and disability policy. Here, invisible harm does not mean wholly unseen harm; it denotes cumulative abuse that remains difficult for victims, social networks, and institutions to identify or translate into action. Constrained response denotes the linked restrictions on recognition and disclosure, evidentiary translation, and safe exit. This conceptual analysis synthesises Chinese law and policy, official statistics, reported judicial and legal-aid cases, and interdisciplinary scholarship. It includes disabled people with long-term physical, sensory, intellectual, or psychosocial impairments and is gender-inclusive, while acknowledging that the available cases and evidence are predominantly about women. We argue that the central problem is structural misalignment: formally universal protection assumes independent recognition, communication, evidence production, mobility, and post-separation care. Real-world cases show how communication barriers, event-centred proof, conflicted guardianship, and unavailable replacement care interrupt the pathway. Structural alignment would make each stage usable through accessible disclosure, pattern-based and assisted evidence, and care-enabled protection, linked by accountable multi-agency coordination. Alignment is necessary for equal access to protection but is not a complete solution to domestic abuse, which also requires prevention, perpetrator accountability, and change in gendered and disability-related power relations. The framework offers a mechanism-based tool for evaluating responses in China and in other settings where care and protection depend heavily on families.

## 1 Introduction

Approximately 1.3 billion people, or 16% of the global population, live with disability (World Health Organization, 2022). China is home to an estimated 85 million disabled people (State Council of the People’s Republic of China, 2021). Many rely on family members for personal assistance, mobility, communication, rehabilitation, or income. Family support can be indispensable, but concentrating care and gatekeeping in one relationship can also create unequal control over daily life and access to the outside world. Psychological domestic abuse in this context is therefore both an interpersonal harm and a public health and equity problem shaped by the organisation of care, the accessibility of services, and institutions’ capacity to recognise non-physical violence.

In this article, psychological domestic abuse means a repeated or patterned use of humiliation, intimidation, threats, isolation, surveillance, manipulation, and coercive control that causes psychological harm or erodes autonomy within a family or analogous domestic relationship. This scope is consistent with China’s Anti-Domestic Violence Law, which includes physical and psychological harm, such as recurrent verbal abuse and intimidation, and with the Supreme People’s Court’s recognition of recurrent humiliation, defamation, threats, stalking, and harassment as psychological violence (Standing Committee of the National People's Congress, 2015; Supreme People's Court of the People's Republic of China, 2022). Verbal abuse is one possible means of psychological abuse; physical and sexual violence are analytically distinct modalities, although they may co-occur with psychological abuse and form part of the same coercive pattern. The present analysis centres psychological abuse and refers to physical or other violence only where it clarifies escalation, evidence, or exit.

The phrase invisible harm is relational rather than absolute. It refers to harm that may be experienced intensely yet remain low in visibility at three levels: the victim may have difficulty naming a normalised pattern; relatives or community members may not witness conduct occurring in private; and institutions may not convert cumulative accounts into recognised evidence or protection. Constrained response describes the resulting reduction in feasible action across three interdependent stages: recognition and disclosure, evidentiary translation, and safe exit and recovery. Neither term implies that disabled people lack agency; both direct attention to how available choices are narrowed by abuse, inaccessible environments, and dependence.

Disabled people are understood in line with the relational approach of the Convention on the Rights of Persons with Disabilities: people with long-term physical, mental, intellectual, or sensory impairments whose interaction with barriers may hinder full and equal participation (United Nations, 2006). The term therefore includes people with psychosocial disabilities associated with severe or enduring mental health conditions when those conditions are long-term and interact with social or institutional barriers. Inclusion does not justify treating diagnosis as evidence of incapacity. Responses for people with psychosocial disabilities may require supported decision-making, trauma-informed communication, continuity of voluntary mental-health care, and safeguards against coercion; people with sensory, physical, or intellectual disabilities may require different communication, mobility, or personal-assistance supports.

The population of interest is disabled people of all genders. Gender is not an eligibility criterion for the framework, but it is an important axis of power and risk: international evidence shows heightened exposure among disabled women (Australian Institute of Health Welfare, 2020; World Health Organization, 2024), and the publicly reported Chinese cases available for this analysis predominantly concern women. The article therefore avoids generalising women’s reported experiences to all disabled people and identifies the lack of evidence about disabled men, gender-diverse people, and variation across impairment groups as a research gap.

We use structural misalignment to describe a mismatch between formally available rights and the capacities or resources that institutions presume a victim can independently exercise. Structural alignment would exist when recognition, evidence, and protection procedures are designed around diverse communication, decision-making, mobility, and care requirements and work as a connected pathway. The article asks: where does the Chinese response pathway become unusable; how do failures accumulate across its stages; and what institutional changes would make protection usable? Its distinct contribution is to integrate barriers often discussed separately in law, welfare, policing, health care, and disability services into a recursive, testable pathway. Alignment is proposed as a necessary condition for equitable protection, not as the ultimate solution to psychological domestic abuse.

## 2 Conceptual approach, source base, and contribution

This article is a conceptual analysis rather than an empirical qualitative study. It synthesises Chinese statutory and policy frameworks, official disability and judicial statistics, publicly reported court and legal-aid cases, and interdisciplinary scholarship on domestic abuse, disability, stigma, and help-seeking. The cases are explanatory instances used to expose institutional assumptions and pathway failures; they are not a representative sample and cannot establish prevalence or causal effects.

The analysis proceeded through a problem-oriented reading of the source base. Materials were organised around three functions that protection systems ordinarily require a victim to perform: identify and disclose abuse, translate experience into institutionally acceptable evidence, and establish a safe alternative to the abusive environment. We then examined how inaccessible communication, discounted testimony, constrained mobility, guardianship conflict, and care dependence can interrupt each function. Goffman’s work on face and interaction, together with scholarship on psycho-emotional disablism, helps connect interpersonal degradation with institutional exclusion.

Existing Chinese law already prohibits psychological domestic violence, permits applications for personal safety protection orders on behalf of some disabled people, recognises multiple forms of evidence, and assigns roles to courts, police, civil affairs departments, women’s federations, disability federations, and community bodies (Standing Committee of the National People's Congress, 2015; Supreme People's Court of the People's Republic of China, 2022). The problem is therefore not the complete absence of relevant mechanisms. It is their uneven accessibility and weak linkage. The framework’s added value is to test whether these distributed mechanisms operate as one usable pathway under conditions of disability and care dependence, and to show how failure at one stage changes the probability of success at the next (see Table 1).

**Table 1:** Key terms, scope, and analytical use.

Term	Definition used in this article	Boundary or analytical role
Psychological domestic abuse	Repeated or patterned psychological harm or erosion of autonomy through humiliation, intimidation, threats, isolation, surveillance, manipulation, or coercive control. Verbal abuse is one possible means; physical and sexual violence are distinct but may co-occur.	Centres cumulative pattern and impact rather than a single visible incident.
Disabled people	People with long-term physical, sensory, intellectual, or psychosocial impairments whose interaction with barriers may hinder full and equal participation (United Nations, 2006).	Includes psychosocial disability; diagnosis alone does not establish incapacity.
Invisible harm	Harm that is difficult to name, witness, document, or recognise institutionally; it is not literally absent or necessarily unknown to the victim.	Specifies three levels of low visibility: personal, social, and institutional.
Constrained response	A narrowing of feasible action across recognition/disclosure, evidentiary translation, and safe exit/recovery.	Describes restricted options, not lack of agency.
Structural misalignment	A mismatch between universal formal rights and institutional assumptions of independent recognition, communication, evidence production, mobility, and care.	Diagnostic concept identifying where a response pathway becomes unusable.
Structural alignment	Accessible and coordinated design that enables diverse victims to use protection at every stage, with supported decision-making, continuity of care, consent, and accountability.	Necessary for equal usability of rights, but insufficient without prevention and accountability.

**Figure 1:**
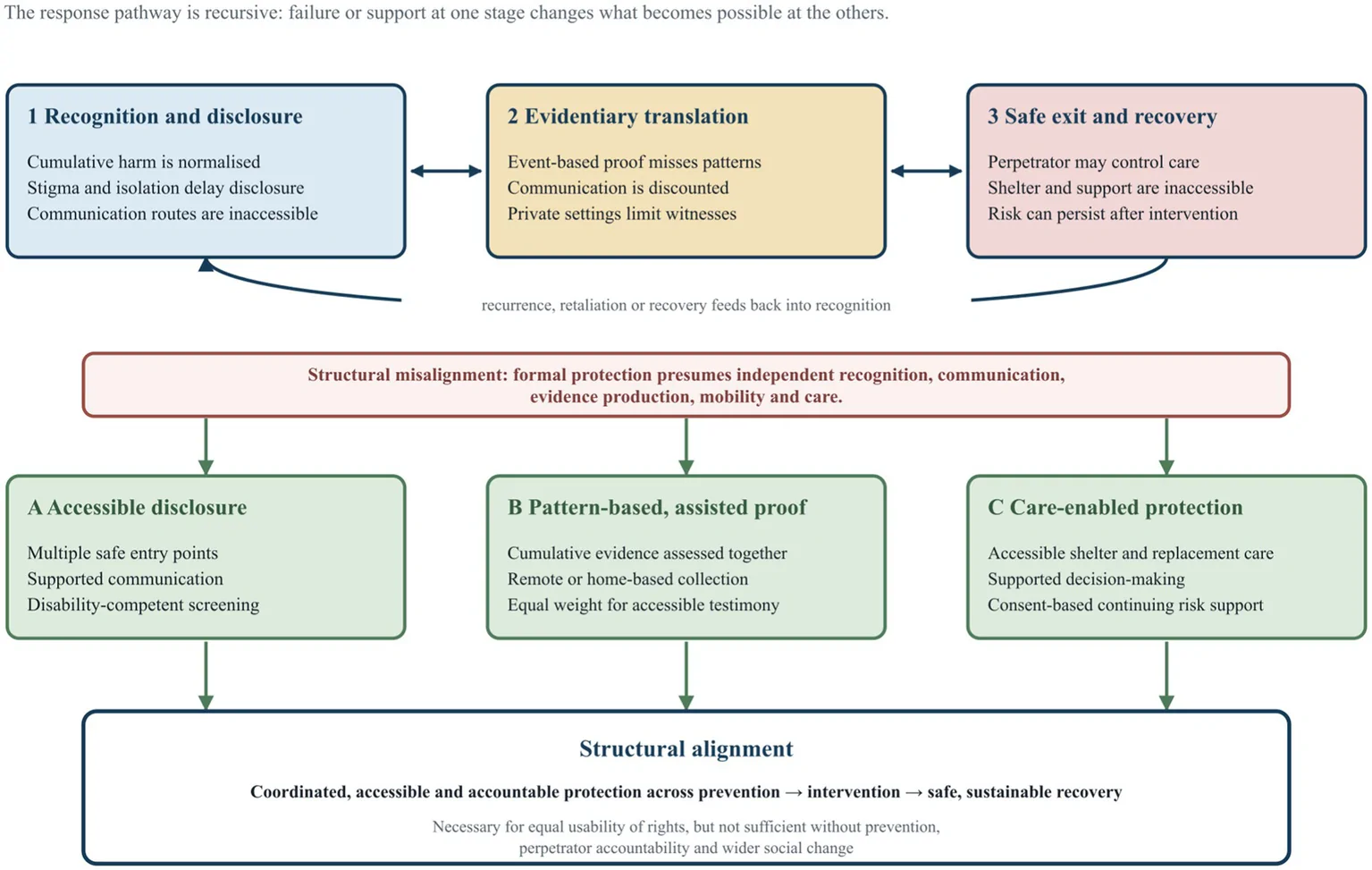
From structural misalignment to disability-inclusive protection. The upper boxes show three recursively linked stages; bidirectional arrows indicate that failure, retaliation, or support at one stage changes what is possible at the others. The lower boxes correspond one-to-one with the aligned functions in Table 2. Structural alignment makes rights usable but does not replace prevention, perpetrator accountability, or wider social change. Original conceptual synthesis by the authors.

The framework is recursive rather than strictly linear. Recognition affects what evidence is preserved; evidentiary recognition affects access to protection; the availability of safe exit affects whether disclosure is feasible; and retaliation or recovery can alter later recognition and help-seeking. Bidirectional arrows in Figure 1 represent these feedback effects. The paired lower boxes identify the aligned institutional function corresponding to each barrier stage; Table 2 reproduces the same one-to-one architecture and anchors it in Chinese examples.

**Table 2:** Framework-to-case-to-response matrix corresponding to Figure 1.

Stage	Institutional presumption	Illustrative Chinese evidence	Aligned response	Safeguard
1. Recognition and disclosure	Victims can privately identify abuse, communicate conventionally, and navigate services.	Huzhou: a deaf woman reported that she did not know police contact or evidence preservation was possible (Tu and Xu, 2025). Tang County: inaccessible, unreliable assistance exposed a deaf woman to fraud (Ministry of Justice Legal Aid Center, 2025).	A. Accessible disclosure: multiple safe entry points, supported communication, disability-competent screening, and confirmed referral.	Consent, privacy, conflict checks for support persons, and no presumption of incapacity.
2. Evidentiary translation	A discrete incident, standard narrative, and official record can adequately establish harm.	Li v. Zhang: reported alternative proof did not secure recognition of psychological abuse (China Judgments Online, 2014). Xuhui data: psychological violence was common among protection-order cases, while evidence preservation remained difficult (Legal Daily, 2025).	B. Pattern-based, assisted proof: cumulative assessment, accessible testimony, remote or home collection, and reasons for evidentiary decisions.	Equal weight for communication formats, trained independent facilitators, and recording of accommodations.
3. Safe exit and recovery	A victim can separate physically and continue daily life without the perpetrator’s care or gatekeeping.	Cang County: the alleged perpetrator was also guardian, requiring capacity and guardianship procedures before divorce (Hebei Rule of Law Daily, 2025). Changle: divorce followed only after livelihood and placement arrangements were secured (Fujian Changan Network, 2025).	C. Care-enabled protection: accessible shelter, replacement assistance, income and health-care continuity, supported decision-making, and follow-up.	Avoid coercive institutionalisation; preserve will and preferences; monitor safety with consent.

Cases are explanatory instances, not a representative sample. The table uses the same three-stage and A–C alignment structure as Figure 1.

This approach has boundaries. Public cases disproportionately represent matters that reached institutions and were considered reportable. Reports may simplify legal reasoning, omit unsuccessful help-seeking, and overrepresent women. Disability groups are heterogeneous, and several sources describe disability services generally rather than domestic-abuse services specifically. The analysis therefore claims mechanism-level transferability, not population representativeness or that every disabled person experiences the same pathway.

## 3 A three-stage pathway of constrained response

### 3.1 Recognition and disclosure

Recognition and disclosure comprise the processes through which a victim names a harmful pattern, communicates it to another person or institution, and seeks support. Psychological abuse can isolate and cognitively disorient victims, making early disclosure difficult (Trafford and Le, 2025). For disabled people, these effects may interact with inaccessible information, dependence on the perpetrator, and prior experiences of being disbelieved. The constraint is therefore relational: it is produced neither by abuse nor impairment alone.

First, abuse can reshape interpretation and self-worth. Repeated devaluation may be internalised as self-blame, while reliance on family care may be reframed as a debt that must be repaid through tolerance. People with intellectual or psychosocial disabilities may receive little accessible information for distinguishing supportive care from coercion, and fluctuating distress can make disclosure harder. These circumstances do not erase agency or credibility. They indicate a need for plain-language information, supported decision-making, repeated opportunities to disclose, and trauma-informed assistance. Experiences reported among people with cerebral palsy likewise show how social expectations and accumulated failure can produce self-imposed limits (Duan, 2023).

The effects of psychological abuse are cumulative and context-dependent. This can interact with pre-existing stigma, making harm appear ordinary and suppressing recognition of the need for help. Research on everyday harm among people with cognitive disabilities shows why analysis must attend to the victim’s lived experience rather than only to a perpetrator’s isolated acts (Idle et al., 2025). Low visibility at the personal level is thus connected to a wider social failure to provide language, validation, and accessible routes for naming harm.

Second, help-seeking may intensify feelings of shame. Publications by the World Health Organization indicate that when violence occurs, disabled women are often placed in situations of extreme isolation, where stigma and discrimination further limit their access to services and information (World Health Organization, 2024). Scholars working at the intersection of disability and affect have emphasised the political significance of shame. Existing research tends to focus either on the negative consequences of impairment and external discrimination or on promoting narratives of disability pride. This tendency positions positive and negative emotions as oppositional, while treating feelings such as shame and inferiority as undesirable deviations from an ideal state (Ingram, 2025).

Drawing on Erving Goffman’s theory of face, face represents the positive social value an individual claims in interaction, and its loss can generate shame, embarrassment, or anxiety (Spiers, 1998). Seeking help constitutes a “face-threatening act,” as it involves acknowledging vulnerability and exposing personal hardship. Although contemporary society increasingly promotes inclusion and support for disabled people, the stigma associated with domestic violence has not been eliminated. For disabled people, disclosing psychological abuse within the family is not merely a legal or protective act; it entails exposing deeply private and vulnerable aspects of their lives.

This disclosure raises multiple concerns. Individuals may worry that their experiences will be overinterpreted, reinforcing stereotypes of disabled people as inherently fragile or unfortunate. They may also fear that revealing family matters will subject both themselves and their relatives to excessive scrutiny and social judgment, creating additional pressure. As Carol Thomas argues through the concept of psycho-emotional disablism, a distinction must be made between “barriers to doing” (restrictions on participation) and “barriers to being” (erosion of self-worth and ontological security) (Thomas, 1999). Psychological domestic abuse operates primarily at the level of “being.” The perpetrator’s repeated devaluation is internalised as self-denial, leading disabled people to view themselves as burdens. Once this internalisation reaches a certain threshold, seeking help is no longer perceived as accessing support, but rather as an admission of failure and inadequacy.

Third, the support pathway itself may be difficult to use. As of 2024, China reported 2,927 legal-aid coordination institutions and 2,699 legal-aid workstations for disabled people, alongside approximately 113,000 staff across disability federations at provincial, municipal, county, and township levels (China Disabled Persons’ Federation, 2025). These figures document an institutional base but do not show how many sites provide domestic-abuse expertise, accessible communication, private disclosure, or rapid referral. They therefore cannot by themselves establish adequacy relative to need.

Beyond the limited number of support channels, both the methods and intensity of legal awareness and outreach also require substantial improvement. In a case from Huzhou, Zhejiang Province, a woman with profound hearing and speech impairments, who had not completed primary education and relied largely on self-study, was subjected to domestic violence by her husband. She was unable to call the police due to communication barriers and could neither cry out nor effectively resist during episodes of abuse. When later recounting her experience to prosecutors, she stated that she “did not know calling the police was an option” and was unaware of the need to preserve evidence or seek medical assessment after abuse (Tu and Xu, 2025). This case illustrates that legal awareness initiatives targeting disabled people remain largely confined to “special projects” rather than forming part of a comprehensive and universal system of public legal education (Shenzhen Disabled Persons’ Federation, 2025).

Of particular concern, even when some disabled people develop the awareness to seek help, the absence of authoritative and accessible support channels may expose them to further harm. In Tang County, Hebei Province, a deaf woman who had endured long-term domestic violence was defrauded of 5,500 yuan by a so-called “legal service company” when attempting to seek assistance. Exploiting her communication barriers and informational asymmetry, the company falsely promised to assist with divorce litigation, resulting in both financial loss and additional psychological distress (Ministry of Justice Legal Aid Center, 2025). This case highlights a critical issue: in the absence of reliable and disability-adapted support systems, help-seeking itself may become a source of risk.

In addition, there is a lack of effective coordination among institutional actors within the existing support system. In a divorce case adjudicated by the Luotian People’s Court, a deaf woman subjected to prolonged domestic violence was unable to pursue legal action independently due to communication barriers (Luotian County People’s Court, 2025). The resolution of her case ultimately required coordinated intervention from the court, procuratorate, public security authorities, and local village committee. While this outcome demonstrates the potential effectiveness of multi-agency collaboration, it also reveals that such coordination remains largely *ad hoc* and case-driven, rather than institutionalised or routine.

For disabled people whose cognitive or communicative capacities may already be constrained, navigating such a fragmented and complex support network presents a substantial barrier. They may not know where to begin, nor are they able to independently manage communication and coordination across multiple institutions. Accordingly, the inadequacy of the help-seeking system is not merely a matter of insufficient resources, but reflects a deeper structural problem in the organisation and accessibility of support itself.

### 3.2 Evidentiary translation and legal recognition

Evidentiary translation is the conversion of lived, often cumulative harm into information that police, courts, health services, or welfare agencies can recognise and act upon. Psychological abuse is often low in external visibility because it occurs privately, may leave no physical injury, and becomes meaningful through repetition and context. The problem is not that psychological evidence is inherently unreliable, but that event-centred procedures can fail to assess patterns.

Chinese law and judicial guidance contain more flexibility than practice may suggest. Article 20 of the Anti-Domestic Violence Law addresses official records, and the Supreme People’s Court’s 2022 provisions list a broad range of relevant evidence, including party statements, police and institutional records, audio-visual materials, electronic communications, medical records, complaints received by disability federations and other bodies, and witness testimony; they also apply a ‘high probability’ standard in personal safety protection order cases (Supreme People's Court of the People's Republic of China, 2022; Wang, 2025). The alignment question is whether victims can access these routes and whether cumulative material is assessed together in practice.

Concerns remain that official police documentation is sometimes treated as functionally indispensable, despite the non-exhaustive evidentiary framework. A review of judgments reported in the existing source base describes claims being rejected for insufficient evidence or reframed as ordinary family disputes even where alternative proof was offered. In Li v. Zhang, the court granted divorce but did not recognise psychological abuse or award damages for emotional harm (China Judgments Online, 2014). This example is illustrative rather than evidence of national prevalence.

Recent developments further illustrate this gap. In 2025, the Supreme People’s Court released typical cases on anti-domestic violence, including two cases in which victims died by suicide following sustained psychological abuse (National Working Committee on Women Children, 2025). In commentary on these cases, a practicing lawyer emphasised that the evidentiary goal in such cases is not to identify a single isolated incident, but to demonstrate the existence of a sustained pattern of coercive and oppressive relations. This requires forms of evidence such as audio recordings, chat histories, documentation of abusive language, and medical or psychological records reflecting the impact of abuse.

However, these forms of evidence often sit uneasily with current evidentiary practices, which remain oriented toward discrete, event-based proof. Even for non-disabled people, gathering such longitudinal and relational evidence demands considerable time, awareness, and resources. For disabled people, who may rely heavily on family members and face barriers in communication or access, these requirements become significantly more burdensome.

This difficulty is also reflected in official data. A judicial white paper on personal safety protection orders issued by the Xuhui District People’s Court in Shanghai reports that, over a nine-year period, 42 out of 69 cases involved psychological violence, accounting for more than 60% of all cases (Legal Daily, 2025). Yet courts consistently encountered problems related to victims’ lack of awareness of evidence preservation and limited capacity to collect relevant materials. These findings underscore a structural tension: while psychological abuse is increasingly recognised in principle, the evidentiary framework remains poorly adapted to its characteristics, thereby impeding effective legal intervention.

Second, private and interactional settings reduce external visibility. A practising lawyer reported that only a small number of psychological-abuse cases encountered over more than a decade were ultimately recognised as domestic violence, attributing this to difficulties of evidence collection, proof, and judicial recognition (Vista Magazine, 2022). Unlike physical violence that may produce visible injury, recurrent humiliation, intimidation, or manipulation often becomes legible only through a longitudinal record. The relevant evidentiary unit is therefore the pattern, not the absence of a single physical trace.

This invisibility is further reinforced by the combined effects of privacy and interaction. Drawing on Goffman’s dramaturgical distinction between front and back regions, the family home may, in relation to public and institutional audiences, function as a back region in which conduct is shielded from those audiences (Goffman, 1959). The dramaturgical approach has also been applied directly to the study of family life and its performative construction (Collett and Childs, 2009). Interactions in such a setting may be accessible mainly to family members, limiting external observation and oversight. When psychological abuse unfolds within this concealed environment, perpetrators may engage in sustained patterns of belittlement, humiliation, and control while victims remain deprived of external witnessing or intervention.

For disabled people, this structural invisibility is further intensified. Existing evidentiary rules in China’s anti-domestic violence framework are designed on the assumption of the general population’s capacities. They neither fully implement the legislative intent of flexible proof standards nor establish differentiated procedures, accessibility support, or assistive mechanisms tailored to disabled people. As a result, individuals with impairments may fail to meet even the baseline evidentiary requirements expected of ordinary victims, placing them at the margins of the recognition system. As noted in a practical handbook on anti-domestic violence work published by a disability service organisation, the closed nature of the family environment makes abuse difficult to detect and evidence difficult to obtain, thereby emboldening perpetrators (A. D. I. Institute for Development Studies, 2023).

Third, barriers differ across impairment groups and cannot be addressed by a single accommodation. Deaf people, people with speech impairments, and people who do not use standard written language may need sign-language interpretation, augmentative communication, or additional time. The problem is not an inability to give evidence as such, but that conventional channels may not receive their expression accurately or may wrongly equate communication difference with lack of intent (Peking University Law Database, 2018).

In one case from Rizhao, Shandong Province, Sun, a woman with profound hearing and speech impairments who was also illiterate and unable to use sign language, was described as “unable to communicate normally, unable to care for herself, and unable to express her intentions.” When she attempted to initiate civil proceedings following the death of her son, the court dismissed her claim on the grounds that it could not confirm whether the lawsuit reflected her genuine intent. Although the factual circumstances of her son’s death were verifiable, she was only able to pursue her rights successfully after receiving legal assistance (Rizhao Municipal Justice Bureau, 2021). This case illustrates how, in situations where harm must be articulated through narrative, such as psychological abuse, communication barriers can prevent victims from transforming lived experiences into legally recognisable evidence. At present, judicial and legal aid institutions have yet to establish professional systems to facilitate accessible communication for disabled people.

People with intellectual or psychosocial disabilities face a related but distinct risk: institutions may discount their accounts because expression is non-linear, communication needs fluctuate, or a diagnosis is wrongly treated as global incapacity. In a reported Changle case, a woman with a severe intellectual disability endured domestic violence for more than two decades and could not independently articulate a legal claim (Fuzhou Political Legal Affairs Commission, 2025). Such cases call for supported communication, repeated and accessible interviewing, and independent assessment of will and preferences, rather than automatic substitution of another person’s decision.

By contrast, individuals with severe physical impairments may possess intact cognitive and communicative abilities, yet remain wholly dependent on perpetrators for mobility and daily functioning. This dependence deprives them of the practical conditions necessary for independent help-seeking and evidence collection. In such cases, the barrier is not epistemic, but material: the absence of autonomy in action effectively forecloses access to both protection and proof.

### 3.3 Safe exit and recovery

Safe exit and recovery refer not only to legal separation but to the ability to remain safe while retaining the assistance, income, housing, communication, health care, and social connection required for daily life. A remedy is formally available but practically ineffective when using it entails loss of essential support.

First, disabled people face substantial barriers in exiting abusive environments. For non-disabled victims, once domestic violence is legally recognised, a range of protective measures becomes available. These include personal safety protection orders that enable physical and spatial separation from the perpetrator, court orders prohibiting further abuse, eviction of the perpetrator from the shared residence, and restrictions on contact or harassment. In addition, administrative warnings, public security penalties, and even criminal sanctions may be imposed to constrain abusive behaviour. Victims may also choose physical separation, such as living apart, thereby accessing multiple pathways to leave the abusive environment and prevent further harm.

For disabled people, however, such pathways are often unavailable in practice. Their daily survival is frequently bound to family members, and the current supply of accessible shelters, professional care services, and temporary placement arrangements remains limited. As a result, even when psychological domestic abuse is formally recognised and legal remedies are granted, victims may still be forced to remain within the same household. In such cases, legal recognition does not translate into actual separation, and protective measures fail to be effectively implemented in lived reality.

Moreover, for many disabled people, the initial step in seeking public legal protection is not simply to prove the existence of abuse, but to overcome additional procedural barriers tied to their legal status. In some cases, this requires initiating special legal procedures, such as a formal determination of civil capacity or the replacement of a legal guardian, before any substantive claim, such as divorce, can even be brought. These procedures are often complex and time-consuming.

A case handled by the Cang County Procuratorate illustrates this difficulty. A woman with an intellectual disability had endured prolonged physical and psychological abuse within marriage. However, her legal guardian was her husband, the perpetrator himself, making it impossible for her to initiate divorce proceedings independently. As noted by the officials involved, under the Civil Code, it was first necessary to assess her civil capacity and then change her guardianship before any further legal action could proceed (Hebei Rule of Law Daily, 2025). This example highlights a deeper structural constraint: the close entanglement between legal status and lived dependency may deprive disabled people of the very capacity to initiate the process of exit.

Second, dependence on family-based care as a structural constraint. Disabled people are often embedded in relationships of survival dependence that differ qualitatively from those of the general population. Their daily living, financial support, mobility, and even basic conditions of survival are frequently reliant on family members. For some groups, independent living is not a realistic option. This structural dependence directly shapes how remedies are applied in practice.

When authorities assess domestic violence cases involving disabled people, they often take into account the victim’s post-separation living conditions. While this consideration appears protective, it can significantly delay access to remedies. In a case adjudicated by the Changle People’s Court, a woman with a severe intellectual impairment had suffered domestic violence for over two decades. Her injuries were documented by her mother through photographs and submitted to the court, which ultimately recognised the existence of abuse. However, the court refrained from granting divorce until it had coordinated with local civil affairs authorities and the disability federation to ensure that the victim would receive subsistence support and be placed in an institutional care facility (Fujian Changan Network, 2025). Only after these arrangements were secured was the divorce finalised.

During the proceedings, judicial officials explicitly noted that the victim’s post-divorce living capacity had to be assessed and that adequate support had to be guaranteed before the case could be concluded. This illustrates a crucial point: for disabled people, legal recognition of abuse does not suffice. Exit from an abusive relationship is conditional upon the prior resolution of livelihood concerns. Accordingly, access to remedies is structurally tethered to the availability of welfare support.

The existing source base does not provide a national count of accessible domestic-abuse shelters, replacement personal assistance, or alternative living arrangements. Official disability-development reports document broader service activity but cannot verify domestic-abuse-specific capacity (China Disabled Persons’ Federation, 2025). Claims about national shortage should therefore be treated as a priority for empirical audit rather than as a quantified finding.

Third, the persistence and escalation of harm after intervention. Psychological domestic abuse is inherently repetitive and concealed. For disabled people, the act of disclosing abuse may itself trigger further harm. The exposure of violence can provoke retaliatory responses from perpetrators, who may escalate abusive behaviour following external intervention. Psychological abuse may intensify into more severe forms, including physical violence, social isolation, and economic control.

Disclosure can increase risk when the perpetrator controls mobility, communication, medication, finances, or personal assistance. Legal intervention without a safety plan may therefore expose a victim to retaliation. Protection orders and warnings can constrain conduct, but their practical value depends on enforceability, safe communication, substitute care, and follow-up. Continuing support should be survivor-centred and consent-based; it should not become routine surveillance of disabled households.

A case from Yangxin County, Huangshi City, illustrates this limitation. Zhang, a woman with an intellectual disability, endured repeated domestic violence over a five-year period and reported the abuse to the police on twelve occasions (Yangxin County People’s Court, 2023). Although local authorities issued a formal warning letter to the perpetrator, ordering him to cease abusive behaviour, the violence continued. Zhang later applied for a personal safety protection order, yet no follow-up information on her subsequent protection was reported. This case highlights a broader systemic issue: existing remedies not only struggle to prevent recurrence, but also lack mechanisms for post-intervention monitoring and support.

## 4 Policy and practice agenda

### 4.1 Accessible recognition, disclosure, and support

Policy reform should be organised around the same three functions shown in Figure 1 and Table 2. The immediate objective is not to create a parallel legal system for disabled people, but to make existing rights usable through stage-specific accessibility, reliable hand-offs, and accountability for completion of the pathway.

Priority 1 is accessible recognition and disclosure. Existing legal-service and disability-service channels should offer multiple confidential entry points, including text, sign-language video, augmentative and alternative communication, and in-person support. Staff need domestic-abuse and disability training, safe identity verification, and protocols for checking whether a proposed support person has a conflict of interest. Information should explain psychological abuse in plain language and distinguish supportive care from coercive control. A single referral coordinator should remain responsible until the receiving service confirms contact.

### 4.2 Pattern-based and disability-inclusive evidence

Priority 2 is pattern-based and accessible evidentiary translation. Reform should operationalise existing flexibility by requiring decision-makers to assess multiple sources cumulatively, document reasons for discounting accessible testimony, and distinguish an isolated family conflict from a repeated pattern of control and harm.

Police records and medical injury reports should not become de facto prerequisites where law permits broader proof. Statements communicated through sign language, writing, symbols, communication devices, plain language, or a trained facilitator should receive equal consideration. Intellectual or psychosocial disability should not be used as a categorical reason to exclude a statement; assessment should focus on the circumstances, support provided, consistency across time, and corroboration available in the full record.

In addition, the scope of admissible evidence for psychological domestic abuse should be explicitly expanded. This may include observations by relatives, neighbours, community workers, and disability service personnel; personal records such as diaries, social media posts, or digital notes; electronic communications from perpetrators, including messages, voice recordings, and chat histories; as well as medical and psychological documentation reflecting the impact of abuse. While any single piece of evidence may be insufficient on its own, courts should recognise patterns of harm where multiple forms of evidence collectively establish a high probability of abuse.

Second, accessible and proactive evidence-collection mechanisms should be developed. Evidentiary systems must move toward disabled people, rather than requiring individuals to adapt to rigid procedural frameworks. At the physical level, home-based evidence collection should be expanded. For individuals with severe mobility impairments or long-term confinement, police and judicial personnel should be authorised to conduct on-site interviews and evidence recording using portable audio-visual equipment. At the technological level, remote evidence collection should be promoted. By integrating disability service hotlines with public legal service systems, digital platforms can be developed to allow victims to provide statements through sign language video, speech-to-text, or other assistive technologies. Such systems can generate time-stamped electronic records, reducing the burden of travel while improving efficiency.

Qualified interpreters and trained communication facilitators should be available throughout evidence collection. A victim may choose an independent trusted person to be present, but services should screen for conflicts because a relative or caregiver may be the perpetrator or gatekeeper. Interviews should preserve privacy, voluntariness, the person’s own words or communication, and an audio-visual record where lawful. For psychosocial disability, support may include breaks, repeated interviews, advance statements, and continuity of voluntary mental-health care without conditioning credibility on treatment compliance.

Finally, clearer standards for recognising psychological domestic abuse should be established. This includes the development of detailed judicial guidelines specifying categories of abusive conduct, evidentiary rules, and the allocation of the burden of proof. Coordinated action by judicial and administrative authorities could provide a unified framework for handling such cases. At the same time, the regular publication and dissemination of guiding cases would help standardise judicial practice across different regions and contexts.

Equally important is the training of legal and law enforcement personnel. Programmes should incorporate disability awareness and accessible communication skills into both initial training and ongoing professional development, enabling frontline actors to identify psychological abuse more effectively and to interpret diverse forms of expression. Public legal education must also be strengthened. Given the limited access to information among some disabled groups, outreach materials should be adapted into accessible formats, including sign language videos and braille publications, and disseminated through targeted channels. Such efforts can help individuals understand what constitutes psychological domestic abuse, how to preserve evidence, and where to seek assistance.

### 4.3 Care-enabled protection and accountable coordination

Priority 3 is care-enabled protection. Temporary accommodation should be audited for physical, sensory, communication, and psychosocial accessibility, while emergency plans should include replacement personal assistance, medication and health-care continuity, income and transport, assistive technology, and support for children or dependants. Where a guardian or caregiver is the alleged perpetrator, independent advocacy and urgent review of guardianship or support arrangements may be required. The source base does not verify whether a dedicated national shelter network is preferable to upgrading mainstream services; comparative evaluation should precede large-scale adoption.

Implementation should connect legal remedies to material survival. Short-term emergency funds, priority access to community support and long-term care, trauma-informed counselling, and employment or social-inclusion services can make separation sustainable. Eligibility and referral rules should be public, accessible, and auditable. Policy should measure completed access, safety, and continuity of care rather than only the number of institutions or referrals.

Coordination is the connective infrastructure of the three-stage pathway. Courts, police, civil affairs departments, health services, women’s federations, disability federations, legal-aid bodies, and community organisations need shared referral protocols, named responsibility, time limits, accessible records, and feedback to the victim. Information sharing should be limited to what is necessary, lawful, and consistent with informed consent and immediate safety.

Community-level actors can support early identification by making optional, accessible outreach and private disclosure available through routine disability and public-health services. Risk assessment should be based on disclosed indicators and professional judgement, not automatic categorisation of all disabled households as risky. Any follow-up must include clear consent, privacy safeguards, a safe way to decline contact, and independent complaint mechanisms. Where immediate serious danger is identified, action should follow the applicable legal safeguarding duties.

Public authorities should avoid downgrading repeated intimidation, humiliation, isolation, or control to ordinary family disputes. Initial responders should document the context and pattern, explain protection-order and legal-aid options accessibly, and make a warm referral rather than merely provide contact information. Warnings or orders should trigger an individualised safety and care plan where the perpetrator controls essential support.

Civil affairs and health services are central to emergency accommodation, replacement care, trauma-informed support, and continuity of treatment. Disability federations and independent advocates can provide communication and navigation support. If a guardian is alleged to have abused the person, any review or replacement process should prioritise the person’s will and preferences, provide independent representation, and avoid unnecessary institutionalisation.

Through mechanisms of information sharing, rapid referral, and joint intervention, such a network can address the limitations of single-agency responses. More importantly, it can provide disabled people with continuous, accessible, and sustainable protection, ensuring that responses to psychological domestic abuse move beyond isolated interventions toward a comprehensive system of care.

## 5 Discussion

### 5.1 What structural alignment can—and cannot—solve

The analysis shifts attention from whether disabled victims make appropriate choices to whether protection systems are usable under conditions of dependency. Recognition, proof, and exit are not separate procedural problems. Internalised stigma or inaccessible information can delay disclosure; delayed disclosure weakens the record; limited evidence reduces access to protection; and the absence of replacement care can make even a successful order unusable. Conversely, knowledge that safe care is available may make disclosure feasible, and supported evidence collection can improve access to protection. The feedback loops in Figure 1 capture this recursive relation.

Structural alignment therefore has a precise but limited role. It means that each stage accepts diverse ways of recognising, communicating, deciding, moving, and receiving care, and that agencies complete hand-offs across stages. It is a necessary condition for equal usability of formal rights. It is not an ultimate remedy for psychological domestic abuse at large. Primary prevention, perpetrator accountability, economic security, anti-stigma work, gender equality, and transformation of coercive family and care relations remain necessary.

### 5.2 Chinese specificity and mechanism-level transferability

Several features of the framework are specifically Chinese: reliance on family-based care; the statutory architecture of the Anti-Domestic Violence Law and personal safety protection orders; roles assigned to disability federations, women’s federations, civil affairs departments, procuratorates, courts, police, village and residents’ committees; and guardianship procedures that may intersect with divorce and protection. Policy recommendations must therefore be implemented through these institutions and evaluated across China’s regional variation rather than assumed to operate uniformly.

What may travel is the mechanism, not the institutional blueprint. In other Asian or comparable societies where care is heavily family-provided, services are fragmented, and victims are expected to disclose and exit independently, the three questions remain useful: Can a person recognise and communicate harm safely? Can a cumulative account become actionable evidence? Can protection be used without losing essential care? Countries with stronger community-based personal assistance, different guardianship law, or more accessible shelters may show a different pattern of failure. Comparative studies should test rather than presume transferability.

### 5.3 Policy priorities, research agenda, and limitations

The immediate policy agenda is to: (i) publish accessible definitions and reporting information; (ii) establish multiple confidential disclosure routes and warm referral; (iii) operationalise pattern-based evidence and accessible testimony; (iv) attach replacement care and livelihood planning to protection; and (v) assign named multi-agency responsibility with consent, privacy, and outcome monitoring. A phased approach should begin with accessibility and capacity audits, pilot the linked pathway in diverse localities, evaluate survivor-defined safety and continuity of care, and scale only interventions that demonstrate benefit without coercion.

The research agenda follows directly from the framework. First, accessible survivor-led qualitative studies should examine how psychological abuse is named across physical, sensory, intellectual, and psychosocial disability and across genders. Second, court, police, legal-aid, health, shelter, and disability-service records should be audited to estimate attrition at each pathway stage. Third, mixed-method and implementation studies should test whether communication support, cumulative evidence protocols, warm referral, and replacement care improve safety. Fourth, comparative research across Chinese regions and Asian care systems should identify which mechanisms are context-dependent. Outcomes should include autonomy, accessibility, safety, recurrence, continuity of care, and unintended coercion, not only reporting or order counts.

Several limitations temper the argument. The source base is documentary and case-based, and no interviews with survivors, caregivers, practitioners, or officials were conducted. Public reporting may omit unsuccessful help-seeking and simplify complex judicial reasoning. The examples predominantly concern women and do not support inference about gender differences. Some numerical indicators describe disability services generally rather than domestic-abuse-specific capacity. The proposed causal links and recommendations are therefore hypotheses for empirical testing, not estimates of effect.

## 6 Conclusion

Psychological domestic abuse against disabled people is not literally invisible; it becomes difficult to name, witness, document, and act upon when cumulative harm meets inaccessible institutions and care dependence. In China, the central barrier is a linked failure of accessible disclosure, evidentiary translation, and care-enabled exit. Structural alignment would make formal rights usable by accepting diverse communication and decision-making, assessing patterns rather than isolated incidents, and preserving care and livelihood during protection. It is a necessary systems response, not a complete cure for domestic abuse. Prevention, accountability, gender equality, economic security, and change in coercive care relations must accompany it. The framework’s practical test is simple: can a person use protection without first demonstrating capacities that the abusive relationship or the institutional environment has denied?

## Data Availability

The original contributions presented in the study are included in the article/supplementary material, further inquiries can be directed to the corresponding author.
